# Mutagenic Tests Confirm That New Acetylacetonate Pt(II) Complexes Induce Apoptosis in Cancer Cells Interacting with Nongenomic Biological Targets

**DOI:** 10.1155/2011/763436

**Published:** 2011-04-10

**Authors:** Sandra Angelica De Pascali, Federica Lugoli, Antonella De Donno, Francesco Paolo Fanizzi

**Affiliations:** Dipartimento di Scienze e Tecnologie Biologiche ed Ambientali, Università del Salento, Prov. le Lecce/Monteroni, 73100 Lecce, Italy

## Abstract

New platinum(II) complexes [PtCl(*O,O′*-acac)(L)] (**1**) and [Pt(*O,O′*-acac)(*γ*-acac)(L)] (**2**) (L = DMSO, **a**; DMS, **b**) containing a single chelated (*O,O′*-acac) (**1**), or one chelated and one *σ*-bonded (*γ*-acac) acetylacetonate (**2**) have been synthesized. The new Pt(II) complexes exhibited high *in vitro* cytotoxicity on *cisplatin* sensitive and resistant cell lines and showed negligible reactivity with nucleobases (Guo and 5′-GMP) but selective substitution of DMSO/DMS with soft biological nucleophiles, such as L-methionine. In order to assess the ability of the new complexes with respect to cisplatin to induce apoptosis by interaction with nongenomic targets, the Ames' test, a standard reverse mutation assay, was carried out on two *Salmonella typhimurium* strains (TA98 and TA100). Interestingly, the new complexes did not show the well-known mutagenic activity exhibited by cisplatin and are, therefore, able to activate apoptotic pathways without interacting with DNA.

## 1. Introduction

After more than 30 years since its first clinical use, *cisplatin* is still one of the most widely used drugs in anticancer chemotherapy [1]. The action mechanism of *cisplatin* has been explained in its essential aspects, relatively to its interaction with DNA. Nevertheless, some essential chemical processes, related to what happens before the *cisplatin* reaches the DNA, generally considered its final target, are still to be identified. Among these processes, the best known is the formation of aquospecies, the main reaction of activation of the drug [2, 3] which occurs in the cytoplasmic compartment by hydrolysis of the chloride ligands. However, many other nongenomic biomolecules could be potential targets for platinum [4, 5]. Sulfur-rich biomolecules, including free amino acids (cysteine and methionine), oligopeptides (glutathione), and proteins represent good targets for a soft metal such as Pt [6–9]. Moreover, the need to improve the *cisplatin* clinical protocol drives much research into better understanding of its antitumor activity mechanism [10]. On the other hand, in order to overcome acquired cellular resistance to *cisplatin*, much effort is currently devoted to the discovery of new Pt anticancer drugs. In the last years several Pt(II) and Pt(IV) complexes have been synthesised, but only a few compounds, such as carboplatin and oxaliplatin [1, 11], are actually used in clinical therapy.

**Scheme 1:**
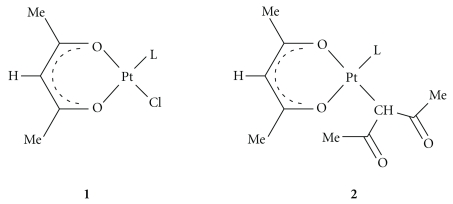
Chemical structure of *O,O′*-chelated acac complexes: **1a **and **2a**, L = DMSO; **1b **and **2b**, L = DMS.

Many studies [12–14], carried out by this research group [15, 16], aimed to understand not only the nuclear, but also the cytoplasmic events taking place in cisplatin-treated cells and able to induce apoptosis. This research group has long been involved in both the synthesis and preliminary evaluation of biological activity of the new Pt complexes and in the subsequent studies of intracellular signal transduction, triggered by these molecules and by *cisplatin* itself [5, 17–19]. Recently, this group has synthesized and studied new platinum(II) complexes containing acetylacetonate (acac) in the metal coordination sphere: [PtCl(*O,O′*-acac)(DMSO)] (**1a**) with only one oxygen-bonded chelate acac (*O,O′*-acac), [Pt(*O,O′*-acac)(*γ*-acac)(DMSO)] (**2a**) containing both an *O,O′*-acac and a *σ*-bonded acac (*γ*-acac) and their dimethylsulphide (DMS) analogues (**1b** and **2b**) having the same key structures (Scheme 1), that have shown interesting biological activities [20–23] and *in vitro* antimetastatic activity [24]. These compounds not only are able to induce apoptosis in endometrial cancer cells (HeLa) [21], with activity up to about 100 times higher than that of *cisplatin*, but also show high cytotoxicity in *cisplatin*-resistant breast cancer cell lines (MCF-7) [21]. The [Pt(*O,O′*-acac)(*γ*-acac)(DMS)] complex (**2b**) with two acetylacetonate ligands, one *O,O′*-chelate and the other one sigma-linked by methine in gamma position, is the more active among the tested complexes [22].

As well as their specific biological activity, these complexes showed an interesting and selective chemical reactivity with nucleophiles with different HSAB (Hard-Soft Acid-Base) character [23]. Indeed, in the complexes [PtCl(*O,O′*-acac)L] (L = DMSO, DMS), containing two ligands with different hard/soft character on the same metal, selective substitution reaction in the presence of further ligands was observed [23]. The more hard ligand replaces the harder one, and the more soft replaces the softer one. However, in these complexes the replacement of Cl^−^ with hard ligands is kinetically and thermodynamically less favoured with respect to the substitution reaction of DMS or DMSO with soft-type ligands. When only a soft ligand is present, as in the case of the complexes [Pt(*O,O′*-acac)(*γ*-acac)L] (L = DMSO, DMS), the reaction takes place only in the presence of soft nucleophile, otherwise there is no reaction [23]. These results, together with the biological studies, indicate that for these complexes, characterized by low reactivity with hard nucleophiles and specific reactivity with soft ligands, the DNA could not be the main target.

In this context further investigations were carried out in order to verify this behaviour. We reported the ^1^H NMR investigations on reactivity of the new compounds with hard and soft biological nucleophiles, such as nucleobases and sulfur amino acids, confirming selective reaction with the latter [23]. In this work the well-known *Salmonella*-*his *reversion test (Ames' Test, a standard reverse mutation assay on the mutagenic capability of the complexes) [25] on two *Salmonella typhimurium* strains, TA98 and TA100, was performed. The bacteria reversed mutation assay (Ames Test), which is normally used to evaluate the mutagenic properties of test substrates, can be also used to assess the ability of tested compounds to interact with DNA [26].

## 2. Material and Methods

### 2.1. Physical Measurements

^1^H NMR spectra were recorded on a Bruker Avance DPX 400, using CDCl_3_, and D_2_O as solvent. ^1^H and ^13^C chemical shifts in CDCl_3_ were referred to TMS, by using the residual protic solvent peaks as internal references. ^1^H and ^13^C chemical shifts in D_2_O were referenced to TSP (2,2,3,3-d(4)-3-(trimethyl-silyl)-propionic acid sodium salt), *δ*(H) = 0 ppm, as an external reference. ^195^Pt chemical shifts were referenced to Na_2_[PtCl_6_] (d(Pt) = 0 ppm) in D_2_O as an external reference [27]. Microanalyses were performed with Carlo Erba Elemental Analyser Mod. 1106 instrument.

### 2.2. Starting Materials

Commercial reagent grade chemicals, acetylacetone, and solvents were used without further purification. [PtCl_2_(DMSO)_2_] [28] and K[PtCl_3_(DMSO)] [29] were prepared according to previously reported procedures.

### 2.3. Syntheses of Complexes

#### 2.3.1. [PtCl(O,O′-acac)(DMSO)] (**1a**)

A solution of acetylacetone (0.097 g, 0.973 mmol) and KOH (0.027 g, 0.487 mmol) in methanol (5 mL) was added dropwise to a solution of K[PtCl_3_(DMSO)] (0.204 g, 0.487 mmol) in water (10 mL) at room temperature with stirring. After few minutes a yellow precipitate separated from the solution. The reaction mixture was left stirring overnight, and the pale yellow precipitate of [PtCl(*O,O′*-acac)(DMSO)] (**1a**) was then isolated by filtration and dried under vacuum (yield 0.149 g, 75%). Anal. Calcd for C_7_H_13_ClO_3_SPt (407.79): C 20.62, H 3.21; found C 20.73, H 3.28.

Alternatively, a solution containing acetylacetone (0.097 g, 0.966 mmol) and KOH (0.027 g, 0.483 mmol) in water (5 mL) was added dropwise to a suspension of *cis*-[PtCl_2_(DMSO)_2_] (0.204 g, 0.483 mmol) in water (10 mL) at room temperature with stirring. The reaction mixture slowly became a yellow solution. After 3 h, a pale yellow solid started to precipitate. The suspension was left under stirring for one day, and the solid was then filtered and dried under vacuum (yield 0.027 g, 26%).

^1^H NMR in CDCl_3_ (298 K): *δ* 2.06s [3H, CH_3_(*O,O′*-acac)], 2.02s [3H, CH_3_(*O,O′*-acac)], 5.56s [1H, CH(*O,O′*-acac)], 3.44s [6H, CH_3_(DMSO), ^3^ *J*_H-Pt_ 40 Hz].

^13^C NMR in CDCl_3_ (298 K): *δ* 26.3 [2C, CH_3_(*O,O′*-acac)], 102.3 [1C, CH(*O,O′*-acac)], 185.1 and 185.9 [2C, CO(*O,O′*-acac)], 44.3 [2C, CH_3_(DMSO)].

^195^Pt NMR in CDCl_3_ (298 K): *δ*  −2399*.*

#### 2.3.2. [Pt(O,O′-acac)(*γ*-acac)(DMSO)] (**2a**)

A solution of acetylacetone (0.358 g, 3.576 mmol) and KOH (0.114 g, 2.860 mmol) in methanol (5 mL) was added dropwise to a suspension of* cis*-[PtCl_2_(DMSO)_2_] (0.302 g, 0.715 mmol) in methanol (20 mL) at room temperature with stirring. The reaction mixture slowly became a pale yellow solution. After one day, the solvent was evaporated under vacuum, and the yellow residue was extracted with CHCl_3_ (10 mL). The chloroform solution was then filtered to remove KCl and K(acac), pentane (30 mL) was added, and the resultant solution was kept overnight at 5°C. Quadrangular pale yellow crystals of [Pt(*O,O′*-acac)(*γ*-acac)(DMSO)] (**2a**) which separated out from the solution were filtered, washed with pentane, and dried under vacuum (yield 0.168 g, 50%). Anal. Calcd for C_12_H_20_O_5_SPt (471.441): C 30.57, H 4.28; found C 30.73, H 4.34.

^1^HNMR in CDCl_3_ (298 K): *δ* 2.00s [3H, CH_3_(*O,O′*-acac)], 1.95s [3H, CH_3_(*O,O′*-acac)], 5.53s [1H, CH(*O,O′*-acac)], 2.29s [6H, CH_3_(*γ*-acac)], 4.79s [1H, CH(*γ*-acac), ^2^ *J*_H-Pt_ 120 Hz], 3.31s [6H, CH_3_(DMSO), ^3^ *J*_H-Pt_ 19 Hz].

^13^C NMR in CDCl_3_ (298 K): *δ* 27.50 and 27.3 [2C, CH_3_(*O,O′*-acac)], 102.2 [1C, CH(*O,O′*-acac)], 185.8 and 184.9 [2C, CO(*O,O′*-acac)], 30.9 [2C, CH_3_(*γ*-acac)], 42.0 [1C, CH (*γ*-acac)], 208.5 [2C, CO(*γ*-acac)], 42.9 [2C, CH_3_(DMSO)].

^195^Pt NMR in CDCl_3_ (298 K): *δ*  −3198*.*

#### 2.3.3. [PtCl(O,O′-acac)(DMS)] (**1b**) and [Pt(O,O′-acac)(*γ*-acac)(DMS)] (**2b**)

To a chloroform (3 mL) solution of** 1a **or **2a **(0.1 g, 0.24 mmol for **1a **and 0.1 g, 0.21 mmol for **2a**) a large DMS excess (0.224 g, 3.6 mmol for **1a **and 0.263 g, 4.24 mmol for **2a**) was added. The reaction mixture was left under stirring at room temperature, overnight. The resulting yellow solution was added to pentane (10 mL) and kept at 5°C for one day up to the formation of yellow needles crystals for **1b **and pale yellow crystals for **2b**. Finally, the crystals were isolated, washed with pentane, and dried under vacuum. (Yield 0.075 g, 0.191 mmol, 80% for **1b**. Anal. Calcd for C_7_H_13_ClO_2_PtS (391.773): C 21.46; H 3.34; found: C 21.27; H 3.20; yield 0.078 g, 0.171 mmol, 82% for **2b**. Anal. Calcd for C_7_H_13_ClO_2_PtS (455.428): C 31.65; H 4.43; found: C 31.72; H 4.56). 

^1^H NMR in CDCl_3_ of **1b** (298 K): *δ* 1.97s [3H, CH_3_(*O,O′*-acac)], 1.88s [3H, CH_3_(*O,O′*-acac)], 5.48s [1H, CH(*O,O′*-acac)], 2.33s [6H, CH_3_(DMS), ^3^ *J*_H-Pt_ 48 Hz]. ^1^HNMR in CDCl_3_ of **2b** (298 K): *δ* 1.89s [3H, CH_3_(*O,O′*-acac)], 1.95s [3H, CH_3_(*O,O′*-acac)], 5.47s [1H, CH(*O,O′*-acac)], 2.20s [6H, CH_3_(*γ*-acac)], 4.88s [1H, CH(*γ*-acac), ^2^ *J*_H-Pt_ 124 Hz], 2.29s [6H, CH_3_(DMS), ^3^ *J*_H-Pt_ 51 Hz].

^13^C NMR in CDCl_3_ of **1b** (298 K): *δ* 26.1 and 26.5 [2C, CH_3_(*O,O′*-acac)], 101.7 [1C, CH(*O,O′*-acac)], 184.9 and 182.9 [2C, CO(*O,O′*-acac)], 22.1 [2C, CH_3_(DMS)].

^13^C NMR in CDCl_3_ of **2b** (298 K): *δ* 27.50 and 27.1 [2C, CH_3_(*O,O′*-acac)], 101.6 [1C, CH(*O,O′*-acac)], 183.7 and 184.7 [2C, CO(*O,O′*-acac)], 30.9 [2C, CH_3_(*γ*-acac)], 40.5 [1C, CH (*γ*-acac)], 207.2 [2C, CO(*γ*-acac)], 22.1 [2C, CH_3_(DMS)].

^195^Pt NMR in CDCl_3_ of **1b** (298 K): *δ*  −2096. ^195^Pt NMR in CDCl_3_ of **2b** (298 K): *δ*  −2905*.*

### 2.4. Reaction of [PtCl(O,O′-acac)(DMSO)], [Pt(O,O′-acac)(*γ*-acac)(DMSO)], and [Pt(O,O′-acac)(*γ*-acac)(DMS)] with Guanosine (Guo), 5′-GMP, and L-Methionine

A solution containing the platinum complex (approximately 2 × 10^−3^ mmol) and an excess of Guo, 5*′*-GMP, or L-methionine (1.6 × 10^−2^ mmol) dissolved in D_2_O (1 mL) was placed in an NMR tube and the reaction monitored by ^1^H NMR spectroscopy. For all complexes tested, the reaction with Guo and 5*′*-GMP was negligible after 24 h, whereas the L-methionine instantly reacted with the initial Pt complexes. 

### 2.5. Mutagenic Test (Ames' Test)

Two strains of *Salmonella typhimurium*, TA98 and TA100 (kindly supplied by Department of Experimental Studies and Applied Medicine, Hygiene Section, University of Brescia), histidine auxotroph mutants, deficient in the synthesis of histidine (*his−*), amino acid necessary for bacterial growth were used according with the method proposed by Ames et al. [25]. These strains contain other mutations that greatly increase the ability to detect mutagens [30]: the mutation “*rfa*” causes partial loss of the lipopolysaccharide barrier coating the surface of the bacteria and increases permeability to large molecules such as benzo[a]pyrene that do not penetrate the normal cell wall; the mutation “*uvrB*” is a deletion of a gene coding for DNA excision repair system, resulting in a high sensitivity to UV rays; the “*R-factor”* plasmid, pKM101, carries ampicillin resistance gene. The histidine auxotrophs will only grow in a medium containing sufficient histidine supplement. To revert to histidine production (prototrophy), or become *his+*, a reverse mutation must occur in the original *his− *mutation site (found in one of the genes involving histidine biosynthesis).

Each sample was investigated with the plate incorporation test: 100 *μ*L/mL of complexes (**1a**, **2a**, and **2b**), at different dilutions with DMSO (from 0.01 *μ*g/plate to 30 *μ*g/plate), were added to 2 mL top agar and to 100 *μ*l of culture of *S*. *typhimurium* growth at optimal concentrations (10^8^ u.f.c/mL). The mix was poured onto a minimal agar plate [30, 31]. Three plates were incubated for each of the dilutions tested. A solvent control (DMSO) was performed. Furthermore, a positive control was carried out using cisplatin, known as compound at strong mutagenic activity [32, 33], testing the same dilutions of new compounds. The results were expressed as numbers of net revertants, calculated as difference between the number of revertants/plate of tested compounds and the number of spontaneous revertants enumerated on the control plates. Moreover, we calculated the mutagenicity ratio (MR)—the ratio of the number of *Salmonella typhimurium* revertants grown in the presence of the tested complex to the number of spontaneously appeared revertants (on the negative control). The sample was considered mutagenic when MR ≥ 2.

## 3. Results and Discussion

### 3.1. Synthesis of Platinum Complexes

**Scheme 2:**
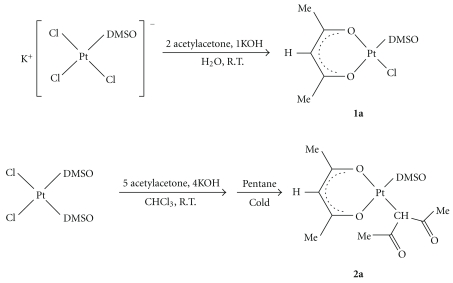
Reaction schemes of **1a** and **2a** syntheses.

The synthesis of [PtCl(*O,O′*-acac)(DMSO)] (**1a**), containing a single chelate acac, was straightforward. Due to its low solubility in the reaction medium, it precipitates as a pale yellow powder from the reaction mixture of K[PtCl_3_(DMSO)] with acetylacetone and KOH in water. Complex **2a **was obtained by treating *cis*-[PtCl_2_(DMSO)_2_] with acetylacetone and KOH in MeOH and was isolated from their respective reaction mixtures by an appropriate workup procedure reported in the experimental section (Scheme 2). 

**Scheme 3:**
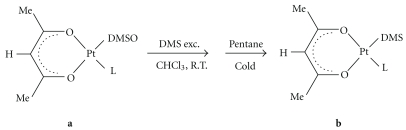
Reaction schemes of **1b** and **2b** syntheses. L = Cl, **1**; L = *γ*-acac, **2**.

In both cases, in order to prevent metal reduction, a slight excess of acetylacetone was used with respect to the calculated stoichiometric amount of KOH based on starting platinum complex. It should be noted that the use of the scarcely soluble *cis*-[PtCl_2_(DMSO)_2_] as a starting platinum complex for the preparation of **1a **in MeOH or water gave unsatisfactory results. Due to the low solubility of *cis*-[PtCl_2_(DMSO)_2_], the reaction with acetylacetone and KOH in MeOH resulted in an excess of acac in solution, even when using a stoichiometric or substoichiometric amount of acac and always gave a mixture of **1a**, **2a** and unreacted starting material. On the other hand, the reaction of *cis*-[PtCl_2_(DMSO)_2_] with acetylacetone and KOH in water, in which both the starting platinum complex and [PtCl(*O,O′*- acac)(DMSO)] are sparingly soluble, gave analytically pure **1a **although a longer reaction time was required and a lower yield was obtained. In the presence of dimethylsulfide (DMS), [PtCl(*O,O′*-acac)(DMSO)] (**1a**) and [Pt(*O,O′*-acac)(*γ*-acac)(DMSO)] (**2a**) complexes selectively undergo substitution of the sulfur ligand to give the analogous DMS complexes [PtCl(*O,O′*-acac)(DMS)] (**1b**) and [Pt(*O,O′*-acac)(*γ*-acac)(DMS)] (**2b**). Interestingly, the substitution reaction appears to be very selective not only for **1a**, where DMSO is the only expected exchangeable ligand, but also for **2a**, where, in principle, the chloroligand was also able to undergo substitution with DMS. Therefore, the synthetic procedures of **1b**-**2b **complexes reported in the experimental section were developed, taking advantage of the selective reactivity showed by **1a**-**2a **compounds towards soft ligand such as DMS. For the synthesis of **1b **and **2b **complexes, an excess of DMS was added to **1a **and **2a**, in order to complete the substitution reaction (Scheme 3).

### 3.2. Reactivity Studies

**Figure 1:**
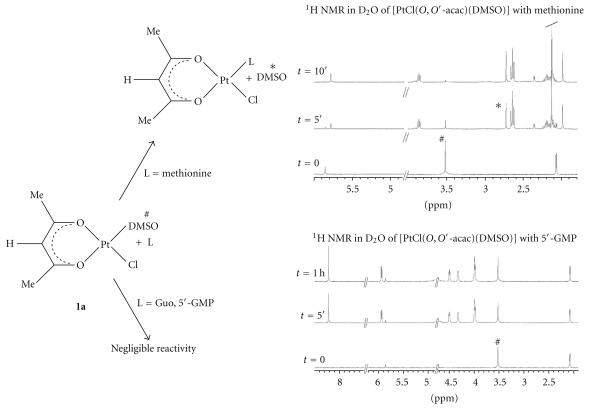
^1^H NMR spectra in D_2_O (400.13 MHz, standard TSP) of **1a** with excess of L-methionine and 5*′*-GMP at different reaction times. Rapid reaction with L-methionine (decreasing coordinated (#) and increasing free DMS (∗) signals) and negligible or very slow reaction with 5′-GMP were observed.

By ^1^H NMR the reactivity of water soluble complexes (**1a**, **2a**, and **2b**) with biological nucleophiles (nucleobases and amino acids) was investigated. The poor water solubility of [PtCl(*O,O′*-acac)(DMS)] (**1b**) prevented further investigation on its reactivity and biological activities. The reactions with soft biological nucleophiles, such as L-methionine (L-met), rapidly gave the same selective substitution of DMSO or DMS, already seen for these complexes towards classical soft nucleophiles (DMS, PPh_3_, ethylene, carbon monoxide) [23]. Indeed, also in the presence of L-methionine excess both **1a** and **2a** complexes gave selective substitution reaction of DMSO affording, respectively, to the neutral species [PtCl(*O,O′*-acac)(L-met)] [Pt(*O,O′*-acac)(*γ*-acac)(L-met)]. Moreover, this substitution reaction not only was more selective but also was faster. In Figure 1 the ^1^H NMR time monitoring of reaction of **1a** with L-met was reported. Adding L-met excess to a solution of **1a** in D_2_O, after only 5 minutes (the time needed to record a ^1^H NMR spectra) the substitution of DMSO ligand and the coordination of L-Met were observed by the decreasing of the signal at 3.44 ppm, assigned to the coordinated DMSO ligand.

**Figure 2:**
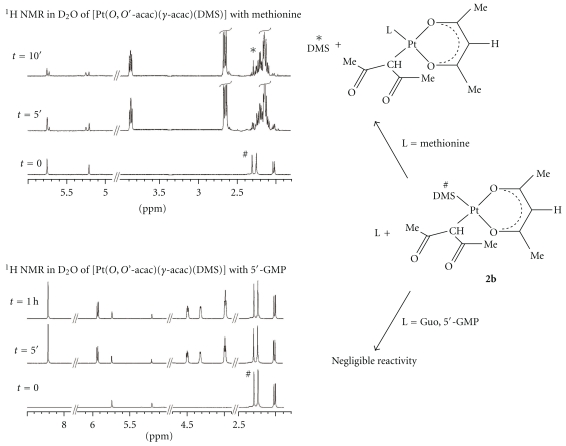
^1^H NMR spectra in D_2_O (400.13 MHz, standard TSP) of **2b** with excess of L-methionine and 5*′*-GMP at different reaction times. Rapid reaction with L-methionine (decreasing coordinated (#) and increasing free DMS (∗) signals) and negligible or very slow reaction with 5*′*-GMP were observed.

At the same time, the increasing of the singlet at 2.6 ppm, attributed to the free DMSO ligand, and the appearance of new signals related to the *O,O′*-acac carrier ligand were detected. After 10 minutes, almost the entire starting complex was reacted with the L-met. The substitution reaction of **1a** and **2a** with soft biological nucleophiles (L-met) was more selective, especially in the case of **1a**, where another good leaving group such as Cl^−^ is present in the coordination sphere. Contrary, in the reactions with biological nitrogen ligands, such as purines (Guo, 5*′*-GMP), both **1a** and **2a** showed little reactivity even after several hours. In the ^1^H NMR spectra in D_2_O of **1a** in the presence of 5*′*-GMP (Figure 1) no new signals of coordinated or free DMSO ligand assignable to substitution reaction products were observed. The same selective reactivity in the substitution of the soft sulfur ligand was observed in the reaction of the water soluble [Pt(*O,O′*-acac)(*γ*-acac)(DMS)] (**2b**) with L-methionine, Guo, and 5*′*-GMP. Also in this case a very fast substitution reaction was noted in the presence of L-met excess. In Figure 2 the ^1^H NMR spectra of reaction of **2b** with L-met were reported.

By addition of L-met excess to a deuterated water solution of **2b**, the decreasing of singlet at 2.29 ppm, attributed to coordinated DMS ligand, and the increasing of new signal of *O,O′*-acac and *γ*-acac, assigned to the [Pt(*O,O′*-acac)(*γ*-acac)(L-met)] species, were identified after only 10 minutes. Analogously to **1a** and **2a** complexes, in the ^1^H NMR time monitoring of the reaction of **2b** with Guo or 5*′*-GMP (Figure 2) any substitution reaction occurred. Such behaviour is very peculiar and suggests a possible selectivity in the substitution reaction at the metal centre in these systems ruled by the hard-soft characteristics of the leaving and incoming ligands. This selectivity could be also operating when the substitution at the metal involves biological sulfur ligands such as thiols or thioethers attached to proteins.

### 3.3. Mutagenic Activity

These complexes have exhibited interesting biological activities [21–23]. Furthermore, these compounds not only induced apoptosis in endometrial cancer cells (HeLa), but also showed high cytotoxicity in cisplatin-resistant breast cancer cell lines, with activity up to about 100 times higher with respect to cisplatin and *in vitro* antimetastatic activity [24]. Among all complexes, [Pt(*O,O′*-acac)(*γ*-acac)(DMS)] (**2b**) was found to be the most active. Differently from cisplatin, for which the activity appears to be associated to both cellular accumulation and DNA linking, intracellular total platinum amount analysis indicated a scarce reactivity of new complexes with DNA, the principal biological target of *cisplatin* [21, 22]. Moreover recent *in vivo* studies aimed to evaluate the outcomes of perinatal treatment with chemotherapeutic agents on key CNS developmental processes such as neural cells proliferation, migration, and differentiation demonstrated that the brain platinum content after [Pt(*O,O′*-acac)(*γ*-acac)(DMS)] treatment was notably higher (approximately 4-fold as much) than after *cisplatin*. However, compared with *cisplatin*, [Pt(*O,O′*-acac)(*γ*-acac)(DMS)] induces less severe changes on fundamental events of neuroarchitecture development [24]. All these data suggested that the cytotoxicity mechanisms of the new complexes may not necessarily require interaction with DNA and that their cytotoxicity is associated only with the intracellular accumulation. The Ames' test, carried out on the new complexes, confirmed these results.

**Figure 3:**
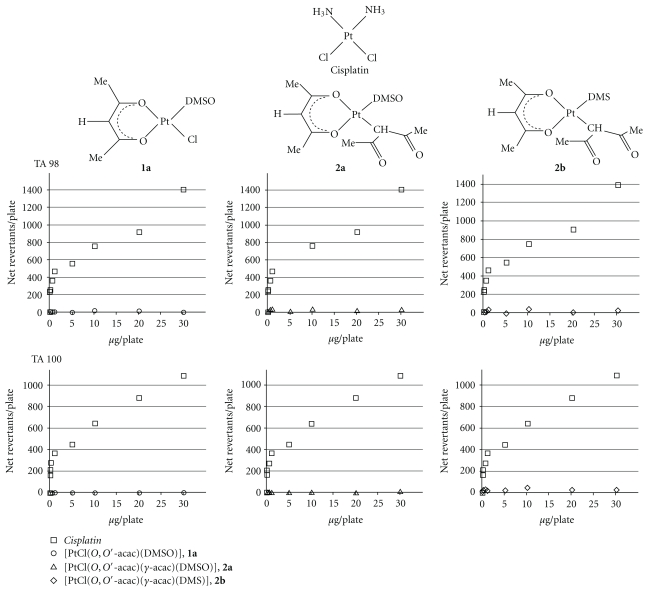
Mutagenicities of **1a**, **2a**, **2b** complexes and *cisplatin* on TA98 and TA100 *Salmonella typhimurium* strains.

The mutagenic activities of new complexes (**1a**, **2a**, and **2b**) with respect to cisplatin on two *Salmonella typhimurium* strains (TA98 and TA100) were reported in Figure 3 and Table 1. As expected a rising of revertants at increasing doses of cisplatin was observed, reaching the highest number of net revertants at the highest tested doses (30 *μ*g/plate) on both strains. Interestingly, at the same tested doses and on both *Salmonella typhimurium* strains the new complexes showed negligible mutagenic activity. Indeed, also at the highest tested doses no net revertants colonies were observed, whereas only *cisplatin* exhibited the well-known [32, 33] dose-dependent increase in revertants. Moreover, for each complex the MR was always below 2.

Hence, we can assert that the biological activity of the new Pt(II) acac complexes is related to the reaction with nongenomic biological targets.

## 4. Conclusions

We have reported new Pt(II) *β*-diketonate complexes with an intriguing chemical reactivity and interesting biological activities. The new complexes coordinate, instead of the mono- and bidentate amine ligands of the classical cisplatin analogues, *O,O′*-acetylacetonate (acac) chelate as carrier ligand and DMS or DMSO ligands. Due to their ability to induce apoptosis in endometrial cancer cells (HeLa) and in cisplatin-resistant breast cancer cell lines (MCF-7) with different pathways with respect to cisplatin, further investigations were performed on the reactivity of novel compounds with biological targets and on the mutagenic capability. Indeed, differently from cisplatin, for which the activity appears to be associated with its intracellular accumulation and formation of DNA adducts, the cytotoxicity of the new compound is only related to the intracellular accumulation.

These complexes, besides their specific biological activity, showed an interesting and selective chemical reactivity towards nucleophile with different HSAB (Hard-Soft Acid-Base) character. The same selective reactivity has been studied towards biological nucleophiles, such as nucleobase and amino acids. The new complexes showed also in these cases negligible reactivity with nucleobases (Guo and 5*′*-GMP) and gave selective substitution of DMSO or DMS with soft biological nucleophiles, such as L-methionine, suggesting that the cellular targets could be amino acid residues in proteins and enzymes involved in the apoptotic induction.

Interestingly, in the mutagenic tests carried out in this work on two *Salmonella typhimurium* strains (TA98 and TA100) the new complexes showed, also at the highest tested doses, insignificant mutagenic activity with respect to cisplatin, known for its strong mutagenic activity and then used as a positive control. All these data suggest that the cytotoxicity mechanisms of the new *β*-diketonate complexes may not necessarily require interaction with DNA and that their biological activity is connected to the reaction with nongenomic biological targets.

## Figures and Tables

**Table 1:** Net revertants/plate (mean ± SD) and MR (mutagenic ratio) of **1a**, **2a**, **2b** complexes and *cisplatin* on TA98 and TA100 *Salmonella typhimurium* strains.

	**1a**				**2a**				**2b**			

	Revertans/plate (mean ± SD)	MR	Revertans/plate (mean ± SD)	MR	Revertans/plate (mean ± SD)	MR	Revertans/plate (mean ± SD)	MR	Revertans/plate (mean ± SD)	MR	Revertans/plate (mean ± SD)	MR
	TA98		TA100		TA98		TA100		TA98		TA100	
Negative control (DMSO)	87.67 ± 16.8		114.67 ± 11.6		82.3 ± 9.6		128.67 ± 21.2		78.7 ± 11.6		119.33 ± 6.4	

Positive control (*cisplatin*) >5 *μ*g/plate	>400		>400		>400		>400		>400		>400	

Dose 1 (0.01 *μ*g/plate)	86 ± 5.6	0.98	62 ± 25.4	0.54	73 ± 1.4	0.89	121 ± 12.7	0.94	66.0 ± 5.6	0.84	141 ± 49.5	1.18

DOSE 2 (0.1 *μ*g/plate)	93.5 ± 3.5	1.07	87 ± 5.7	0.76	99.0 ± 1.4	1.15	119.5 ± 2.1	0.93	97.5 ± 9.2	1.24	144.5 ± 3.5	1.21

DOSE 3 (0.5 *μ*g/plate)	93.5 ± 0.7	1.07	88 ± 0	0.77	108.0 ± 14.1	1.31	134 ± 5.7	1.04	100 ± 1.4	1.27	156.5 ± 9.2	1.31

DOSE 4 (1 *μ*g/plate)	90 ± 1.4	1.03	75.9 ± 9.2	0.66	118.0 ± 2.8	1.43	132 ± 8.5	1.03	116.5 ± 3.5	1.48	141 ± 24	1.18

DOSE 5 (5 *μ*g/plate)	79 ± 2.8	0.90	98 ± 13.4	0.85	98.0 ± 5.6	1.19	132.5 ± 2.1	1.03	81.5 ± 9.6	1.04	142 ± 16.9	1.19

DOSE 6 (10 *μ*g/plate)	106 ± 19.8	1.21	103 ± 29.7	0.90	122.0 ± 2.8	1.48	134.5 ± 3.5	1.05	125.5 ± 31.8	1.60	165 ± 9.9	1.38

DOSE 7 (20 *μ*g/plate)	101.5 ± 14.8	1.16	111 ± 1.41	0.97	103.5 ± 12.0	1.26	116 ± 5.7	0.90	89 ± 4.2	1.13	146 ± 1.4	1.22

DOSE 8 (30 *μ*g/plate)	14.5 ± 0.7	0.17	146.5 ± 21.9	1.28	114.5 ± 7.78	1.39	140 ± 4.2	1.09	109 ± 4.2	1.39	146 ± 19.8	1.22
